# Modern Sociology

**Published:** 1897-01-23

**Authors:** 


### MODERN SOCIOLOGY.

### BABY-FARMING IN FRANCE.

BABY-FARMING is in France a more respectable and better organised industry than here. There it is the rule rather than the exception for families in even respectable circumstances to board their children out—sometimes in the country, sometimes with a wet-nurse in the neighbourhood—for the first two years of their lives. The wet-nurse is a national institution, and you may see these self-important dames any day on the Paris boulevards dressed in a glorified peasant costume, with yards upon yards of gorgeous ribbon twisted round the cap. But it is only the rich who can afford to keep a wet-nurse—always a domineering and exacting personage—on the premises. Those in moderate circumstances send their children away during the time when they are most literally, in our English phrase, encumbrances. The establishment of *crèches* is doing something to destroy this old-established fashion. Working mothers—and in France the wife who works is common in a higher social grade than here—are glad to have their children kept during the day, and to reclaim them at night, and thus keep up the parental relation. It is rather hard to see one's child turn from one and run to another woman; yet if that other woman has brought him up it must be expected. In spite of *crèches*, however, there are, according to some fairly recent statistics, over 18,500 Parisian children whom their parents have desired to be *placés en nourrice*—boarded out with wet or dry nurses.

Over this boarding-out the State exercises a paternal supervision. The nurses, wet or dry, are licensed, and are subject to inspection both by doctors and by lady visitors, who are more likely to note defects in the house or in the general appearance of the child than even a medical man. Deficiency in care or kindliness, in respectability of conduct or suitability of abode may result in the nurse's licence being taken from her. The Prefect of Police also recommended that no licence to take in boarded-out infants should be granted to women over sixty years of age, as these are often obstinately fixed in old-fashioned ideas. He admits, however, that this might often be a hardship to respectable women who, too old for other work, are yet able to bring up a child, and often develop a grandmotherly tenderness for it. More reasonable is the suggestion that a *nourrice au biberon*, *i.e.*, a woman who desires children to bring up "on the bottle," as we say, should not be allowed to take more than one nursling. Dr. Lacronique, the medical inspector who recommended this limitation, brought forward various reasons in support of it, the chief being the reciprocal infection which might result from the same bottle being used by several children. He suggests, however, that the rule might be modified in so far as to permit of a second nursling being taken when the first is six months old, and therefore about to be gradually weaned. In our opinion the six months' regulation is of very little use if it is meant to prevent the use of the same bottle by different children; a nine months' limit is the smallest that should be allowed, and we think that in the restriction to only one nursling lies the best protection against the abuses of the baby-farm, the promiscuous huddling and neglect, better even than nurses' licences and doctors' visits of inspection. In any case, however, the French system is superior to our no-system. Perhaps this is because the children are more generally precious in their parents' eyes.

With us the farmed-out baby is usually the child of shame; the father wants to hear nothing of it, and the mother as little as possible. In France this is not so. Among those with which the statistics we quote are concerned, the legitimate are three times as numerous as the illegitimate. The nurse might therefore expect surprise visits from parents who would not be slow to note any apparent lack of health, cleanliness, and general well-being in the nursling. This possibility would, it is true, be modified by distance from the capital, for of the 18,500 young Parisians whom we are considering only 1,800 were placed out in the Department of Seine. The general reason for sending such an enormous proportion of these grass-orphans (if we may coin the word) so far from home is that the nurses' charges are higher near the capital. This is to be regretted, for, as we have said, the parents' supervision is the child's best safeguard.

In view of the greater benefit received by the child, it is comprehensible why wet-nurses are better paid than others. Yet, looked at from another point of view, there is a certain unfairness in this, for the wet-nurse is put to almost no expense for her nursling, while the dry-nurse must, or ought to, buy a considerable quantity of good milk every day. As a matter of fact, she often does not. Either she waters the milk unduly, or, more often, begins too soon to feed the child on more or less solid food, which can be got cheaper than milk. The fault, for the same reasons of economy, is frequent enough among mothers of the poorer classes, so we need not think too badly of the nurses. To meet this danger, the Prefect of Police suggests that, in Paris at least, the municipality might provide at certain milkshops milk of good quality to be sold at a fixed low price to nurses and to poor families. We doubt if this would entirely remedy the evil, though it might help, especially as the municipality would provide only tested country milk, whereas at present nurses may in ignorance buy the milk of town cows, kept all their life in stalls, and inferior in quality to that of cows which feed on fresh grass in the fresh air. It is to be noted, however, to the credit of dry-nurses, that the deaths among their charges show a tendency to diminish, though they are still greater than among those children who are brought up on Nature's food. Yet the difference is not greater than might take place in any home. The deaths altogether average a little under 10 per cent. of the children under municipal inspection, and of these about 53 per cent. are infants brought up on the bottle.

In fact, though we are by no means inclined to advise the boarding-out of children who have homes, we admit that in so far as it is necessary that it should be done, it is one of those things which they manage better in France. That, in fact, a French child, boarded away from home, for reasons of health or convenience, has a care taken of his welfare by the Government for which an English child, similarly circumstanced, would look in vain.

